# Regional Regulation of Transcription in the Bovine Genome

**DOI:** 10.1371/journal.pone.0020413

**Published:** 2011-06-03

**Authors:** Arun Kommadath, Haisheng Nie, Martien A. M. Groenen, Marinus F. W. te Pas, Roel F. Veerkamp, Mari A. Smits

**Affiliations:** 1 Animal Breeding and Genomics Centre, Wageningen UR Livestock Research, Lelystad, The Netherlands; 2 Animal Breeding and Genomics Centre, Wageningen University, Wageningen, The Netherlands; University of North Carolina at Charlotte, United States of America

## Abstract

Eukaryotic genes are distributed along chromosomes as clusters of highly expressed genes termed RIDGEs (Regions of IncreaseD Gene Expression) and lowly expressed genes termed anti-RIDGEs, interspersed among genes expressed at intermediate levels or not expressed. Previous studies based on this observation suggested a dual mechanism of gene regulation, where, in addition to transcription factors, the chromosomal domain influences the expression level of their embedded genes. The objectives here were to provide evidence for the existence of chromosomal regional regulation of transcription in the bovine genome, to analyse the genomic features of genes located within RIDGEs versus anti-RIDGEs and tissue-specific genes versus housekeeping and to examine the genomic distribution of genes subject to positive selection in bovines. Gene expression analysis of four brain tissues and the anterior pituitary of 28 cows identified 70 RIDGEs and 41 anti-RIDGEs (harbouring 3735 and 1793 bovine genes respectively) across the bovine genome which are significantly higher than expected by chance. Housekeeping genes (defined here as genes expressed in all five tissues) were over-represented within RIDGEs but tissue-specific genes (genes expressed in only one of the five tissues) were not. Housekeeping genes and genes within RIDGEs had, in general, higher expression levels and GC content but shorter gene lengths and intron lengths than tissue-specific genes and genes within anti-RIDGES. Our findings suggest the existence of chromosomal regional regulation of transcription in the bovine genome. The genomic features observed for genes within RIDGEs and housekeeping genes in bovines agree with previous studies in several other species further strengthening the hypothesis of selective pressure to keep the highly and widely expressed genes short and compact for transcriptional efficiency. Further, positively selected genes were found non-randomly distributed on the genome with a preference for RIDGEs and regions of intermediate gene expression compared to anti-RIDGEs.

## Introduction

Eukaryotic genes are not randomly distributed along chromosomes but organised as clusters of highly expressed genes, termed RIDGEs (Regions of IncreaseD Gene Expression), and lowly expressed genes, termed anti-RIDGEs [1], interspersed among genes expressed at intermediate levels or not expressed. Certain genomic features observed for RIDGEs were in striking contrast with those for anti-RIDGEs [2]. RIDGEs were found to be gene dense, GC rich and SINE repeat rich and mostly harboured genes with shorter than average intron sizes. In contrast, anti-RIDGES showed low gene density, were AT rich and LINE repeat rich. Surprisingly, the gene expression patterns of highly and weakly expressed chromosomal regions were roughly similar in all 12 human tissues analysed [1], [2]. Based on their findings, Versteeg *et al.* [2] postulated that RIDGEs globally govern the expression levels of their embedded genes and that this higher level regulation of gene transcription was dependent on factors that act on chromosomal domains like chromatin conformation and position in the nucleus. The existence of this novel domain wide transcription regulatory mechanism, in addition to the well-known regulatory mechanism at the individual gene level that involve transcription factors and regulatory sequences on the gene, was proven in a later study by Gierman *et al.* [3]. They showed that the expression levels of identical green fluorescent protein (GFP) reporter constructs integrated at several different chromosomal positions corresponded to the overall expression level of genes within the domains of integration. To explain this, a dual mechanism of gene regulation was suggested wherein transcription factors determine a basal level of transcription for a gene, whereas the chromosomal domain in which the gene was located determined its ultimate expression level. They also established a range for the sizes of such chromosomal domains by showing that the correlation between GFP expression and domain activity was highest for window sizes of roughly 19–79 genes around the integration sites.

The observation of clusters of highly expressed genes was reported to be a consequence of the clustering of housekeeping (HK) genes, which in turn, was probably the outcome of selective pressure to position widely expressed genes in genomic regions where the higher-order chromatin structure allows better accessibility to the transcription machinery [4], [5]. However, RIDGEs are not restricted to HK genes, but encode tissue-specific (TS) genes too [2]. Moreover, not all genes on a RIDGE are highly expressed but the average expression level of all genes per cluster taken together would be higher than the average gene expression across the genome. Nevertheless, RIDGEs consist of a higher proportion of HK genes than would be expected by chance and the HK genes share certain genomic features with genes on RIDGEs such as higher expression levels and shorter lengths for introns, genes and coding sequences [6], [7]. It has been proposed that since transcription is an energy expensive process, there is an evolutionary selection pressure for economy of transcription to keep widely and highly expressed genes short and compact [6], [8], [9], [10].

The phenomenon of domain wide regulation of transcription has been shown to exist in several species: mammals (human, mouse) [1], [2], [3], [11], [12], flies (drosophila) [13], [14] and recently in birds (chicken) [6]. Though believed to be a feature for all eukaryotes, this has been experimentally verified only in a few species. The recently published bovine genome assembly [15] allowed us for the first time to investigate the existence of chromosomal regional regulation of transcription in bovines using a brain transcriptome dataset of closely related tissues: Amygdala (AM), Hippocampus (HC), Dorsal Hypothalamus (DH), Ventral Hypothalamus (VH) and Anterior Pituitary (AP). Prior to this study, it was not known whether the phenomenon of chromosomal regional regulation of transcription existed in bovine genome and whether the genomic features for RIDGEs and anti-RIDGEs reported in earlier studies in other species could be expected in bovines as well. It was also not known whether the above mentioned findings could be expected when closely related tissues are analysed as was done here, which is in contrast to previous studies that used gene expression data from a wide variety of tissues.

A study on expression levels of genes subject to positive selection [16] showed that positively selected genes, in general, had reduced expression levels and were expressed in a TS manner in several human tissues. Targets of positive selection have been linked to complex trait phenotypes in humans and primates where adaptation to local environmental changes has been the driving force. Likewise, in laboratory, farm and companion animals, human intervention in the form of domestication and intensive artificial selection may have been additional drivers [16], [17], [18], [19], [20]. Therefore the quest for genes controlling complex traits of interest in domesticated animals may be supported by finding signatures of positive selection and regional patterns of gene expression that help narrow down the regions on the genome to look for these genes.

The primary objectives of this study were: 1. to provide evidence for the existence of chromosomal regional regulation of transcription in the bovine genome, 2. to analyse the genomic features of genes located within RIDGEs versus anti-RIDGEs and TS genes versus HK, and 3. to examine the genomic distribution of genes subject to positive selection in bovines.

Here, HK refers to genes expressed in all five tissues studied here and TS refers to genes expressed in only one of the five tissues.

## Results

### Gene expression data

**Figure 1:**
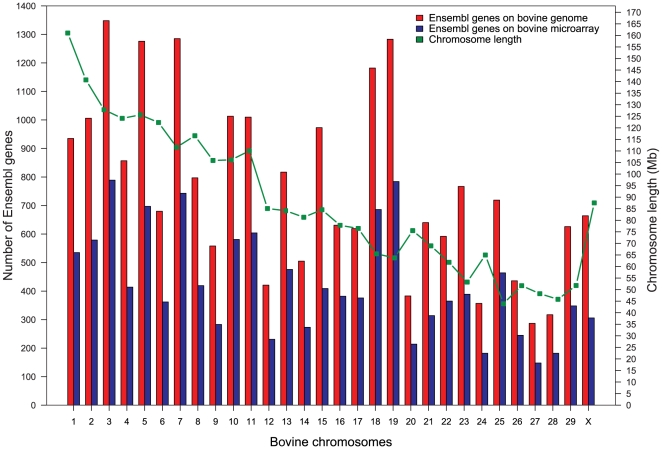
Chromosome wise distribution of Ensembl genes on bovine genome and microarray. The number of Ensembl genes on each chromosome of the bovine genome and those represented on the microarray are depicted by bar plots. The chromosome length is overlaid on this plot.

The normalised transformed gene expression data matrix analysed here consisted of average expression per tissue for 13,234 bovine Ensembl genes (Table S1). Prior to averaging, a hierarchical cluster analysis of the normalised gene intensities per individual per tissue showed clustering by tissue type. Among the brain areas, the AM and HC clustered closer to each other and so did DH and VH, whereas the AP stood out as a separate cluster (Figure S1). The chromosome lengths and chromosome wise distribution of all bovine Ensembl genes and of those bovine genes on the BOMC array represented by good quality probes (see Materials and Methods section) are shown in Figure 1. The number of Ensembl genes represented on the re-annotated BOMC array was roughly 50% of the total known Ensembl genes on each chromosome. The total bovine genome size analysed in this study was 2,608,296,415 bp which was approximately 91% of the latest bovine genome assembly version Btau_4.0 [15], [21] having an estimated genome size of 2.87 Gb. Chromosomes 3, 5, 7, 18 and 19 have the most genes, with over 1100 each. The bovine chromosomes 18 and 19 (BTA18 and BTA19), though ranked 21^st^ and 23^rd^ respectively by length, are ranked fifth and third respectively by the number of genes harboured. We used the “Synteny Tracker” tool [22] to identify syntenic regions between the bovine and human genomes. The high gene density of these bovine chromosomes corresponds to the high gene density of their syntenic human chromosomes. BTA18 shared a large syntenic region with HSA19 which has the highest gene density amongst human chromosomes [23] and also with HSA16 which is of moderate gene density [24]. Likewise, BTA19 was almost entirely syntenic with HSA17 which has the second highest gene density amongst human chromosomes [25].

### Genomic regions of high and low gene expression identified

**Figure 2:**
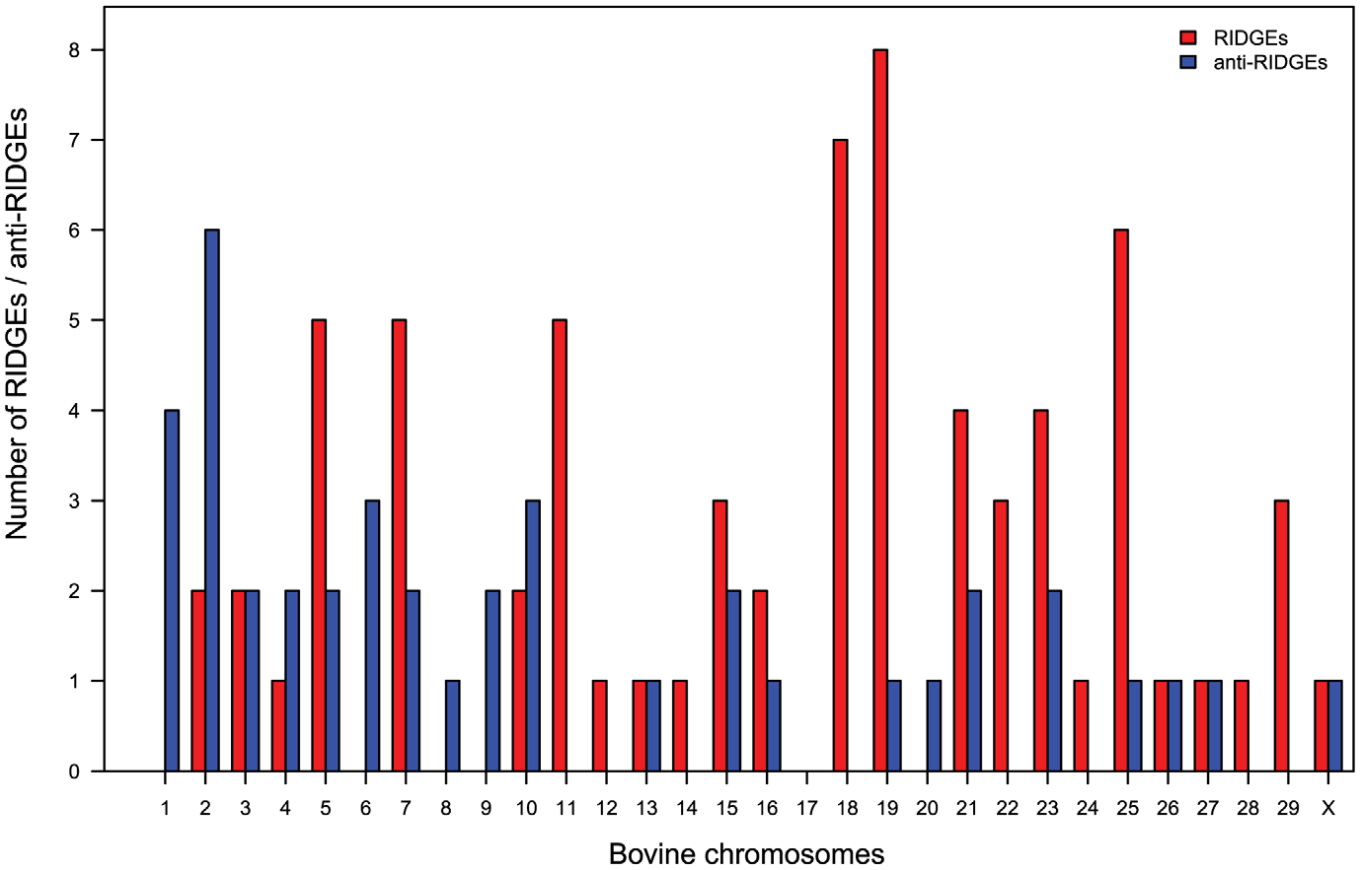
Chromosome wise distribution of RIDGEs and anti-RIDGEs. The number of RIDGEs and anti-RIDGEs found per chromosome (based on median gene expression with a window size of 39 genes) is depicted here.

RIDGEs and anti-RIDGEs were so selected that each covers approximately 10% of the bovine genome (see Materials and Methods section). For our dataset, this criterion was satisfied by taking the expression thresholds as 1.25 times larger than the genomic median expression value in the case of RIDGEs and 1.45 times lower than the genomic median expression value for anti-RIDGEs. With the chosen window size of 39 genes and genome coverage threshold of 10%, a reasonable number of RIDGEs and anti-RIDGEs could be identified: 70 RIDGEs harbouring 3735 bovine Ensembl genes and 41 anti-RIDGEs harbouring 1793 bovine Ensembl genes. The chromosome wise distribution of these RIDGEs and anti-RIDGEs is shown in Figure 2 and their genomic locations on the bovine genome are provided in Table S2. The chromosome wise transcriptome maps based on median expression depicting the identified RIDGEs and anti-RIDGEs are given in Figure S2.

### Higher number of RIDGEs and anti-RIDGEs found than expected by chance

A permutation test repeated 10000 times using the same window size (39 genes) and threshold for RIDGE identification (1.25 times larger than the genomic median expression value) as used in our analysis showed that there was only about a 10% chance for obtaining an equal or higher number of RIDGEs (mean = 60.13, s.d. = 7.07) than what was found in our analysis (n = 70). A similar test for anti-RIDGEs revealed that there was less than 1% chance for obtaining an equal or higher number of anti-RIDGEs (mean = 24.44, s.d. = 4.92) than what was found in our analysis (n = 41).

### Transcriptome maps in four brain areas and anterior pituitary are highly correlated

**Figure 3:**
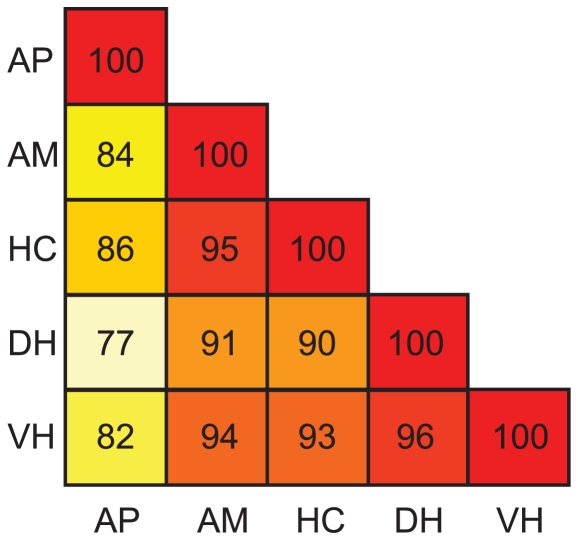
Pair wise correlations between tissues based on median expression values of all genes per tissue. AP-Anterior Pituitary, AM-Amygdala, HC-Hippocampus, DH- Dorsal Hypothalamus, VH-Ventral Hypothalamus.

**Figure 4:**
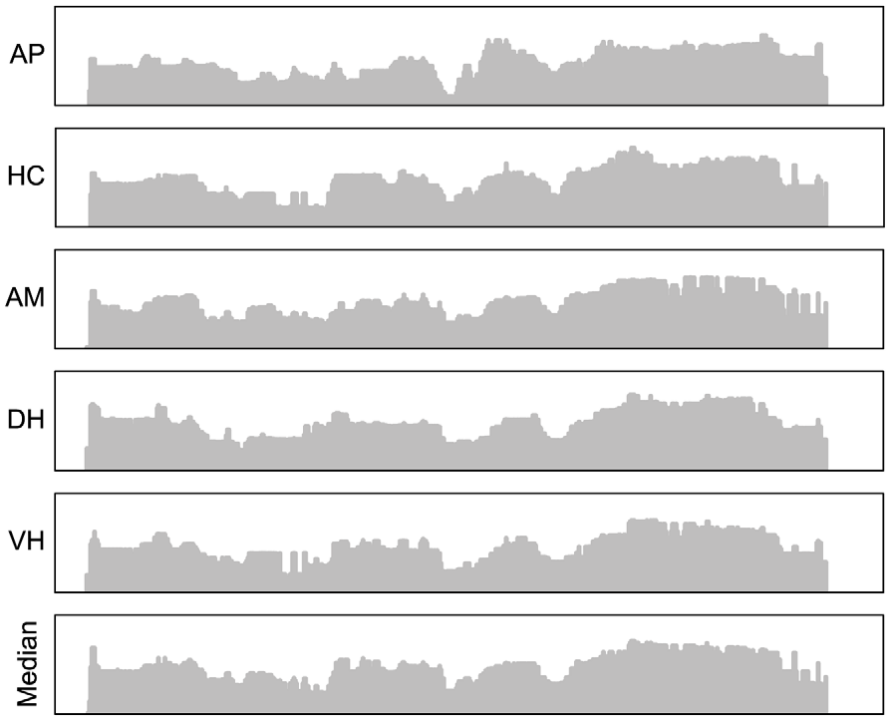
Tissue wise transcriptome maps for chromosome 2. Transcriptome maps for AP, HC, AM, DH, VH and one based on the average of the median gene expressions across all 5 tissues. The y-axis shows the median gene expression levels (log transformed normalised gene intensities ranging from 9 to 14) for the 579 Ensembl genes on chromosome 2 represented on the array (AP-Anterior Pituitary, AM-Amygdala, HC-Hippocampus, DH- Dorsal Hypothalamus, VH-Ventral Hypothalamus).

Spearman rank correlation test on pair-wise comparisons of transcriptome maps of all tissues showed high correlations (Figure 3), with an average correlation of 0.91 (all p-values below 2.2e-16). The highest correlations are between DH and VH and between AM and HC. All brain areas are more correlated with each other than with the anterior pituitary. The similarity in the transcriptome maps across tissues was clearly visible taking chromosome 2 as an example (Figure 4).

### “Housekeeping” and “Tissue-specific” genes identified

**Figure 5:**
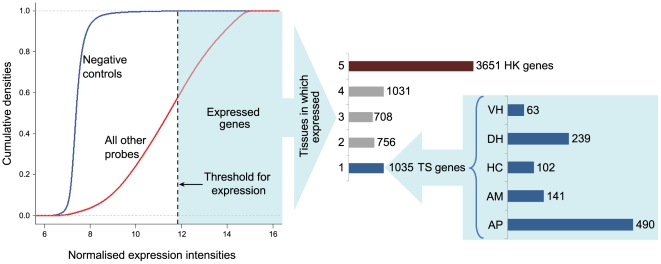
Determination of expressed genes and their tissue wise distribution. The blue line in the graph on the left represents expression levels of negative controls on the microarray and the red line represents that of all other probes. The 99.9% quantile value of expression levels of all negative controls across all arrays was 11.94, which was taken as the threshold above which a gene was considered as expressed. The graph on the right depicts the number of tissues in which the expressed genes are distributed and their tissue wise distributions. Here, ‘1’ represents the “tissue-specific” genes i.e. expressed only in one tissue (total of 1035), ‘2’ represents genes expressed in 2 tissues and so on. Number ‘5’ represents genes expressed in all five tissues which we defined here as “housekeeping” genes (total of 3651). (AP-Anterior Pituitary, AM-Amygdala, HC-Hippocampus, DH- Dorsal Hypothalamus, VH-Ventral Hypothalamus).

In order to define the threshold for expression, the normalized expression intensities of all genes and negative controls across all arrays and tissues was determined (Figure 5). The threshold for expression defined as the 99.9% quantile value of the log transformed expression levels of negative controls across all arrays, was 11.94. Figure 5 also shows the number of tissues in which the expressed genes are distributed, where ‘1’ represents the TS genes expressed only in one tissue (total of 1035 for the five tissues), ‘2’ represents genes expressed in 2 tissues and so on. Number ‘5’ represents genes expressed in all 5 tissues which we defined here as HK genes (total of 3651). The distribution of the 1035 TS genes across individual tissues is also shown (see Table S3 for list of HK and TS genes). The maximum number of TS genes was seen for the AP (490) which is an endocrine gland. The brain areas, being all of neurological tissue type, share a lot of functions in common and therefore genes. Hence the number of unique TS genes found were smaller (141 for AM, 102 for HC, 239 for DH, 63 for VH). Functional analysis of the orthologous human genes of the bovine genes (human orthologues used for better annotation information available) in R package ‘GOstats’ (see Materials and Methods) revealed enriched gene ontology and KEGG pathway terms for the HK and TS genes (Table S4). In most cases, the terms were not significant at a Benjamini-Hochberg p-value threshold of 0.1, hence the top 10–15 terms were considered to get an indication of the most important functions. Briefly, functional analysis of TS genes per tissue revealed processes like ‘negative regulation of synaptic transmission, glutamatergic’, ‘negative regulation of transmission of nerve impulse’ and ‘regulation of behaviour’ for the anterior pituitary, and processes like ‘androgen receptor signalling pathway’, ‘neurotransmitter catabolic process’, ‘behavioural response to pain’ for the brain areas. For HK genes, general processes related to ATP synthesis, mitotic activity and translation, typical of most tissues, were found to be enriched.

### Housekeeping genes are over-represented on RIDGEs but tissue-specific genes are not

Based on 10,000 random samples, the mean percentage overlap between the randomly sampled genes (equivalent in number to the total HK genes) and the RIDGE genes was found to be 17.55% (s.d. = 0.53). However, the overlap between actual HK genes found in our analysis and the RIDGE genes was 23.28% (850 out of 3651 HK genes) which was clearly above expectation (p<0.01). Similarly, the mean percentage overlap between the randomly sampled genes (equivalent in number to the total TS genes) and the RIDGE genes, was found to be 17.52% (s.d. = 1.14). However, the overlap between actual TS genes found in our analysis with RIDGE genes was found to be 13.14% (136 out of 1035 TS genes) which was clearly below expectation (p<0.01).

### Differences observed in several genomic features of RIDGE versus anti-RIDGE genes and “Housekeeping” versus “Tissue-specific” genes

**Figure 6:**
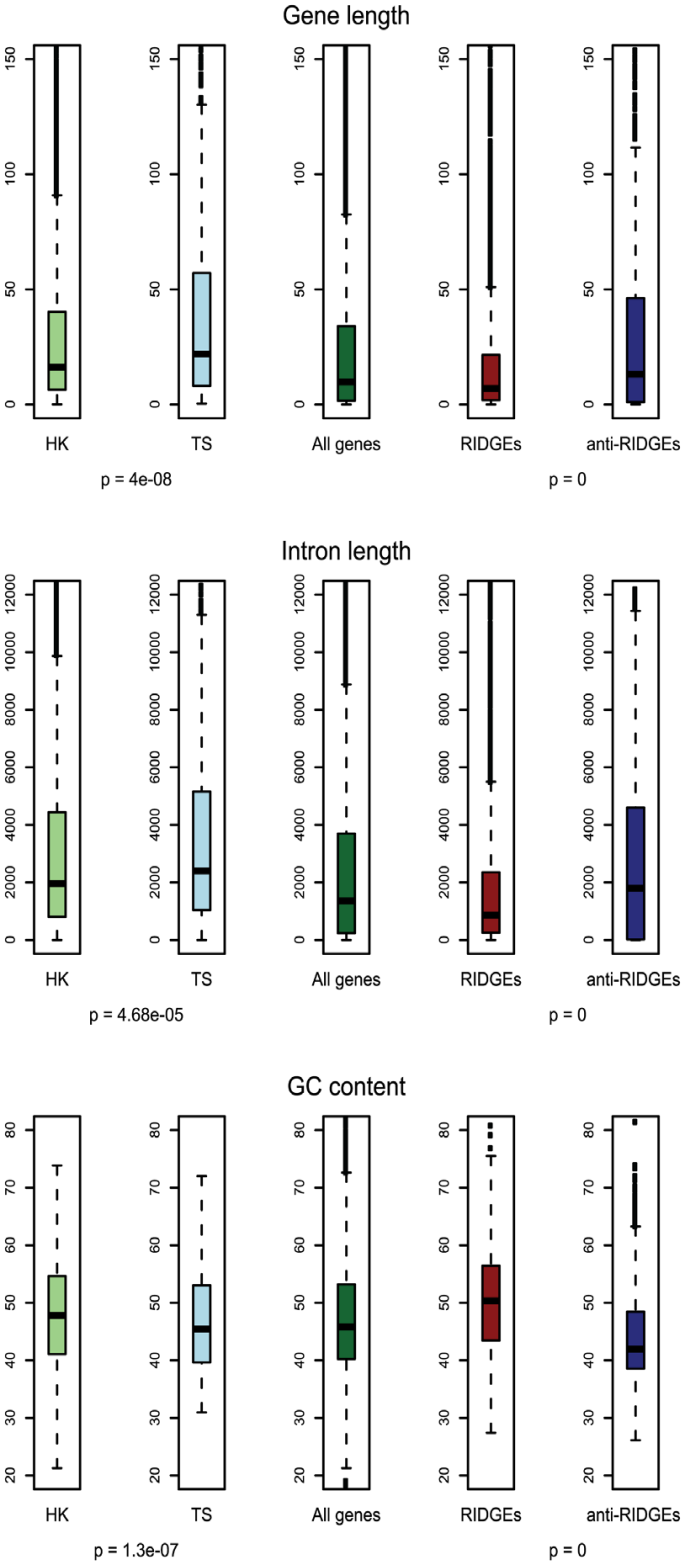
Genomic features of housekeeping vs. tissue-specific genes and of genes present on the RIDGEs vs. anti-RIDGEs. The following genomic features are represented here: Gene length, Intron length and GC content. The p-values of the significance of difference between the genomic feature comparisons are given below each pair of box plots separated by a box plot depicting the feature for all genes together. The bottom and top of the box are represents the 25th and 75th percentile (the lower and upper quartiles, respectively), and the band near the middle of the box represents the 50th percentile (the median). The ends represent the lowest datum still within 1.5 IQR of the lower quartile, and the highest datum still within 1.5 IQR of the upper quartile. Any data not included between the ends are plotted as an outlier with a dot. (HK – housekeeping genes, TS – tissue-specific genes).

The gene length and average intron length were significantly lower in HK genes compared to TS genes and also significantly lower in genes located on RIDGEs compared to anti-RIDGEs. In contrast, the GC content was significantly higher in HK genes compared to TS genes and also significantly higher in genes located on RIDGEs compared to anti-RIDGEs (Figure 6). Further, transcript length and exon count were significantly lower in HK genes compared to TS genes whereas these were not significantly different between genes on RIDGEs compared to anti-RIDGEs. Differences in exon length were non-significant in both cases (Figure S3). The results of these comparisons are summarized in Table 1.

**Table 1:** Comparisons of genomic features of housekeeping vs. tissue-specific genes and of genes on RIDGEs vs. anti-RIDGEs.

	Genomic features	Housekeeping vs. Tissue-specific genes	RIDGE vs. anti-RIDGE genes
1	Gene length	Lower*	Lower*
2	Intron length	Lower*	Lower*
3	GC content	Higher*	Higher*
4	Transcript length	Lower*	Non-significant
5	Exon count	Lower*	Non-significant
6	Exon length	Non-significant	Non-significant

*p<0.01.

### Genes subject to positive selection are not randomly distributed on the genome

Out of the 71 genes reported to be subject to positive selection [21], 54 could be mapped to bovine Ensembl genes with a known genomic location. Of these 54 genes, 12 were found to be located within RIDGEs whereas only one was within an anti-RIDGE (Table S5). This suggests that genes under positive selection are not randomly distributed on the genome (p<0.01) but are less likely to be within anti-RIDGEs i.e. more likely to be found within RIDGEs and regions of intermediate gene expression.

## Discussion

Hierarchical clustering of the arrays showed that arrays clustered based on tissue type but not on days (day0 and day12). The AP being an endocrine gland is clearly different from the brain tissues and this was evident in the clustering results. Among the brain areas, the AM and HC tended to cluster together which may be explained by the close physical and functional proximity between these tissues in the brain. Similarly, the DH and VH are closer to each other physically and functionally as they share many nuclei.

A number of regions of high and low gene expression along the chromosomes were identified. The probability to find as many regions of high and low gene expression as found in our analysis purely by chance is below 10% for RIDGEs and below 1% for anti-RIDGEs, suggesting that RIDGEs and anti-RIDGEs are not a random feature but have an underlying purpose. Given the fact that our data is based on only about 13,000 genes corresponding to good quality probes on the array, we expect more significant results if we had greater number of genes under study. Therefore, even the relatively high 10% chance for RIDGEs may be considered to reflect the trend that RIDGEs are not a random feature in bovine genome. Moreover, the high correlations among the transcriptome maps from the five tissues studied here suggest that regional differences in transcription are a general trend in the bovine genome. High correlations in the global transcriptome profile between diverse tissues have been reported earlier in other species [8].

HK and TS genes were also identified and the fact that HK genes were significantly over-represented on RIDGEs indicates that most of these genes are expressed at a relatively high level. The observations on genomic features of RIDGE versus anti-RIDGE genes and of HK versus TS genes are in agreement with previous studies on human, chicken and other vertebrates. Hence this study extends these general observations also to bovines. The high degree of correlation between gene expression levels and genomic features may indicate an evolutionary adaptation for energy efficiency of transcription by keeping genes which are highly and widely expressed, short and compact [6], [8], [9], [10].

Analysis of the TS genes for enriched gene ontology terms revealed neurological processes as expected for TS genes. For the HK genes, even though our definition was based on only 5 tissues of which 4 were of neurological type, processes enriched among those genes were general processes related to energy metabolism, cell division, transcription etc. expected of most tissues and cells as they are HK functions. However, it is not possible to conclusively prove that all these genes as truly HK in the absence of information from other diverse tissues and physiological conditions of expression. Hence the HK genes reported here, as defined earlier, only represent genes expressed in all brain tissues represented here and the anterior pituitary.

RIDGEs were suggested to be preferred sites for formation of chromosomal aberrations, development of gene deregulation as in tumours and evolutionary breakpoints in mammalian evolution [26]. Here, we found that positively selected genes were preferentially distributed on RIDGEs and regions of intermediate gene expression compared to anti-RIDGEs. This finding seems to contradict that in an earlier study [16], where it was shown that positively selected genes had reduced expression levels and were expressed in a TS manner in several human tissues. However, it may be noted that selection pressures acting on bovines could be quite different than those in humans, particularly because of the effects of domestication and intensive artificial selection in bovines for traits preferred by humans. For example, positively selected genes in bovines include a number of immune related genes which probably arose in response to the substantial rumen microbial load or due to keeping cattle in herds where rapid disease transmission is a persistent threat [21]. Other points to note while comparing our results with those of other studies which may be based on a large number of diverse tissues is that our definition of HK and TS genes are based on only five closely related tissues and that HK genes as defined in our study could overlap with brain-specific genes of other studies.

To conclude, our findings suggest the existence of chromosomal regional regulation of transcription in the bovine genome. The HK and TS genes reported here represent a useful resource for further studying bovine brain expressed genes. The genomic features observed for genes within RIDGEs and HK genes in bovines agree with previous studies in several other species further strengthening the hypothesis of selective pressure to keep the highly and widely expressed genes short and compact for transcriptional efficiency. Another striking observation was that positively selected genes were non-randomly distributed on the genome with a preference for RIDGEs and regions of intermediate gene expression compared to anti-RIDGEs.

## Materials and Methods

### Ethics Statement

The study was approved by the Animal Care and Ethics Committee of the Animal Sciences Group of Wageningen University and Research Centre, Lelystad (Approval ID 2006087a).

### Microarray experiment description and analysis

The microarray experiment was carried out as part of a study aimed at identifying and studying genes that contribute to differences in oestrous behaviour expression and fertility levels of dairy cows [27]. Briefly, oestrous behaviour was recorded in 28 healthy Holstein Friesian cows from 30 days in milk (DIM) onwards till their time of sacrifice which varied between 77 and 139 DIM i.e. after at least 2 oestrous cycles. Samples from 4 brain areas (dorsal hypothalamus, ventral hypothalamus, amygdala and hippocampus) and the anterior pituitary were collected from these cows, 14 of which were sacrificed at start of oestrus and 14 at mid of oestrous cycle. The cows were euthanized in a stress-free, quick and standardized way and all efforts were made to minimize suffering.

RNA extracted from brain tissue samples were hybridized on Bovine 24K oligonucleotide (70-mer) microarrays designed and produced by the Bovine Oligonucleotide Microarray Consortium (BOMC), USA (http://www.bovineoligo.org/). The procedures followed for tissue collection, RNA isolation and microarray hybridization were as described in our earlier study [27]. Briefly, a total of 280 arrays (i.e. 14 cows×2 phases×5 tissues×2 for dye swaps) were prepared in a common reference design with the dye labels swapped between individual samples from each brain area and a reference sample consisting of equal proportions of RNA from all tissues of all cows. Microarray data pre-processing and analysis was done using the LIMMA (Linear models for microarray data) package [28] within Bioconductor project [29] of R statistical programming language (http://www.r-project.org). Briefly, background correction was performed using LIMMA's ‘normexp’ method (with an offset of 50) followed by within-array ‘print tip loess’ normalisation and between-arrays quantile normalisation (‘Aquantile’ method). We then transformed the normalized data, converting M-values back to normalised intensity values so that we could perform an intensity based analysis rather than ratio based. The final gene expression data matrix analysed here was obtained by averaging the intensities of the red and green labelled samples per individual per tissue, then averaging the median gene expression across individuals per phase, and finally averaging the expression values for genes represented by multiple probes on the array.

All microarray experiment data is MIAME compliant and has been deposited in ArrayExpress (http://www.ebi.ac.uk/microarray-as/ae/) (accession number: E-TABM-916). The original annotation provided by BOMC for the bovine 24K oligonucleotide microarray dates back to June 2007. For our analysis, we used the bovine oligonucleotide array probe re-annotation (Version 5) based on Ensembl (http://www.ensembl.org) release 56 (October 2009) provided on the EADGENE website (http://www.eadgene.info/ToolsResources/EADGENEOligoSetsAnnotationFiles/tabid/324/Default.aspx) by the authors of the oligo-set re-annotation pipeline, sigReannot [30]. For the re-annotation, out of the 23,496 probes (excluding control probes) on the bovine oligonucleotide array, only 16,620 probes that were assigned a quality score between 1 and 4 for their specificity to hits on the bovine genome were considered. Probes with quality scores between 5 and 7 had either no hits or multiple hits and were not annotated as they were not specific.

### Identifying genomic regions of high and low gene expression

Similar to the protocol followed by Nie *et al.* [6] for identifying regions of high and low gene expression levels, we first calculated the gene expression levels along the chromosome at the position of every gene on it using moving medians based on a window size of 39 genes i.e. the median of the expression level of that gene and that of 19 genes flanking it on either side. The Robust Scatter Plot Smoothing technique (function ‘runmed’ in R package ‘stats’) was used to calculate the moving median gene expression. The chosen window size of 39 genes falls within the range of sizes established for chromosomal domains [3] and is similar to the size used in earlier studies [1], [2], [6]. We then defined RIDGEs and anti-RIDGEs as those regions on the chromosome that contain contiguous stretches of at least 10 gene positions where the moving median gene expression level at each gene position is a certain fold threshold higher or lower than the overall genomic median expression for the bovine transcriptome [1], [2], [3], [6]. These higher and lower fold thresholds were identified by testing a range of thresholds to select the ones that resulted in the identification of RIDGEs and anti-RIDGEs to cover about 10% of the genome each. As a consequence, the gene expression fold thresholds are not symmetric. The choice of 10% coverage is arbitrary and we could have chosen other realistic genome coverage thresholds above or below this level too, provided enough genes are selected from RIDGEs and anti-RIDGEs to support the study of the genomic features for genes in these regions. Here, we chose 10% genome coverage threshold to maintain uniformity with previous studies [6], [26] and to provide a reasonable number of genes for further study. The rationale for using a genome coverage threshold, in addition to expression fold threshold, was to ensure that the total region studied is constant for both RIDGEs and anti-RIDGEs. In the absence of any prior knowledge on the presence and extent of coverage of RIDGEs and anti-RIDGEs on bovine genome and given the fact the there are several criteria that could affect the size of the RIDGEs and anti-RIDGEs, we set the genome coverage threshold to a constant value in order to make the study consistent. In addition, the criteria of genomic coverage thresholds will make the methodology used in this study scalable to any microarray platform or species.

### Testing the probability of finding RIDGEs and anti-RIDGEs by chance

We know from studies in other species that RIDGEs and anti-RIDGEs represent a higher order structure in the genome and there is a non-random distribution or clustering of genes based on their expression levels [1]. Here, after identifying RIDGEs and anti-RIDGEs with the criteria we defined, we tested whether the numbers of RIDGEs and anti-RIDGEs identified in bovines were indeed greater than that expected by random chance. The number of regions identified would be a function of the region size which depends on criteria we defined i.e. a combination of thresholds for gene expression level, genome coverage, window size and contiguous stretch of gene positions satisfying the gene expression threshold. Nie *et al.* [6] showed that with window sizes of 29, 39 and 59, the number of RIDGEs identified reduced proportionately but it was still significantly higher in the actual genome than on a randomised genome based on permutations of gene positions. Here too, we do the random permutation test to test whether, with similar settings, there would be an over-representation of regions with overall high or low gene expression on the actual genome compared to the randomised genome. In brief, the test involved permuting the genomic locations of Ensembl genes on the genome and repeating the RIDGE/anti-RIDGE analysis 10,000 times to create 10,000 random transcriptome maps and then comparing the number of RIDGEs/anti-RIDGEs thus identified to the number identified in the actual analysis.

### Testing the correlation between tissue-specific transcriptome maps

To test for pair wise correlations among the transcriptome maps on all the chromosomes (applied to the running median expression values with window size of 39 genes) across tissues, the non-parametric Spearman correlation test was used on the ranks of the paired transcriptome maps.

### Identifying ‘Housekeeping’ and ‘Tissue-specific’ genes and their functional analysis

We defined a threshold level for expression as the 99.9% quantile value of the expression levels of all negative controls on the array. Genes expressed above this level specifically in one tissue alone were defined as TS genes and those expressed in all five tissues were defined as HK genes. In order to test whether the identified TS genes and HK genes contained genes with specific functions expected of these categories, a functional analysis was done using R package ‘GOstats’ [31]. To take advantage of the better annotation available for human genes, we first converted the cattle genes to their orthologous human genes. Genes in the study sets were tested for enriched Gene Ontology (GO) [32] terms and KEGG [33] pathway terms. The population set against which the study set genes were tested consisted of the Ensembl IDs of the orthologous human genes, of which 11,589 remained after removing duplicates.

### Testing RIDGEs for over-representation of housekeeping or tissue-specific genes

For testing if HK genes are over-represented on RIDGEs, we first randomly sampled the same number of genes from the total Ensembl genes on the array as the number of HK genes found in our analysis. We repeated this sampling procedure 10000 times and for each random sample, we calculated the percentage overlap of the sampled genes with the genes found to be present on the RIDGEs. We performed a similar procedure to test if TS genes are over-represented on RIDGEs. Based on these, we derived the distributions of expected percentages of HK genes and of TS genes on RIDGEs under the null hypothesis. We compared this with the actual percentage of HK genes and TS genes found on RIDGEs in our analysis.

### Genomic features of RIDGE versus anti-RIDGE genes and “Housekeeping” versus “Tissue-specific” genes

Similar to the protocol followed by Nie *et al.* [6], genomic features of RIDGEs versus anti-RIDGEs and HK versus TS genes were determined and the significance of the differences between each pair for all features were statistically tested using Wilcoxon rank-sum test (function ‘*Wilcox.test*’ in R package ‘*stats*’). The features compared were: gene length (genomic length), intron length (averaged intron length of all transcripts per gene), GC content, transcript length, exon count and exon length. The information required for these calculations was retrieved from the Ensembl genome database using BioMart [34] on 1^st^ September 2010.

### Genomic distribution of genes subject to positive selection in bovines

To analyse the relation between chromosomal regions of differing gene expression levels and genes under positive selection, we examined the genomic distribution of 71 bovine genes previously identified as being subject to positive selection [21]. Using Fisher's Exact Test, we tested whether the positively selected genes have a non-random pattern of distribution on the genome with respect to RIDGEs, anti-RIDGEs or regions of intermediate gene expression levels.

## Supporting Information

Figure S1 **Hierarchical clustering of microarrays.** Hierarchical cluster analysis of the gene expression intensities for all individuals showed that arrays from the same brain area tended to cluster together but the effect of day was not evident. (AP-Anterior Pituitary, AM-Amygdala, HC-Hippocampus, DH- Dorsal Hypothalamus, VH-Ventral Hypothalamus).
(PDF)

Figure S2 **Chromosome wise transcriptome maps depicting the identified RIDGEs and anti-RIDGEs.** The y-axis shows the median gene expression levels (log transformed normalised gene intensities ranging from 9 to 14) for the Ensembl genes on each chromosome represented on the array. The solid red and blue lines depict the RIDGEs and anti-RIDGEs respectively whereas the dotted red and blue lines represent the expression threshold to qualify as RIDGE or anti-RIDGE respectively.
(PDF)

Figure S3 **Genomic features of housekeeping vs. tissue-specific genes and of genes present on the RIDGEs vs. anti-RIDGEs.** The following genomic features are represented here: Transcript length, exon count and exon length. The p-value of the significance of difference between the genomic feature comparison is given below each pair of boxplots separated by a boxplot depicting the feature for all genes together. The bottom and top of the box are represents the 25th and 75th percentile (the lower and upper quartiles, respectively), and the band near the middle of the box represents the 50th percentile (the median). The ends represent the lowest datum still within 1.5 IQR of the lower quartile, and the highest datum still within 1.5 IQR of the upper quartile. Any data not included between the ends are plotted as an outlier with a dot. (HK – housekeeping genes, TS – tissue-specific genes).
(PDF)

Table S1 **Expression data of 13,234 genes in five bovine tissues.**
(XLS)

Table S2 **Genomic locations of RIDGEs and anti-RIDGEs on the bovine genome.**
(XLS)

Table S3 **List of housekeeping and tissue-specific genes.**
(XLS)

Table S4 **Enriched Gene Ontology and KEGG pathway terms in the lists of housekeeping and tissue-specific genes.**
(XLS)

Table S5 **Genomic locations of genes subject to positive selection and their overlap with RIDGEs/ anti-RIDGEs.**
(XLS)
