# Supplementary figures and images for: Regional Regulation of Transcription in the Bovine Genome
Source: PLoS One. 2011 Jun 3;6(6):e20413. doi: 10.1371/journal.pone.0020413 (PMC3108615; doi:10.1371/journal.pone.0020413)

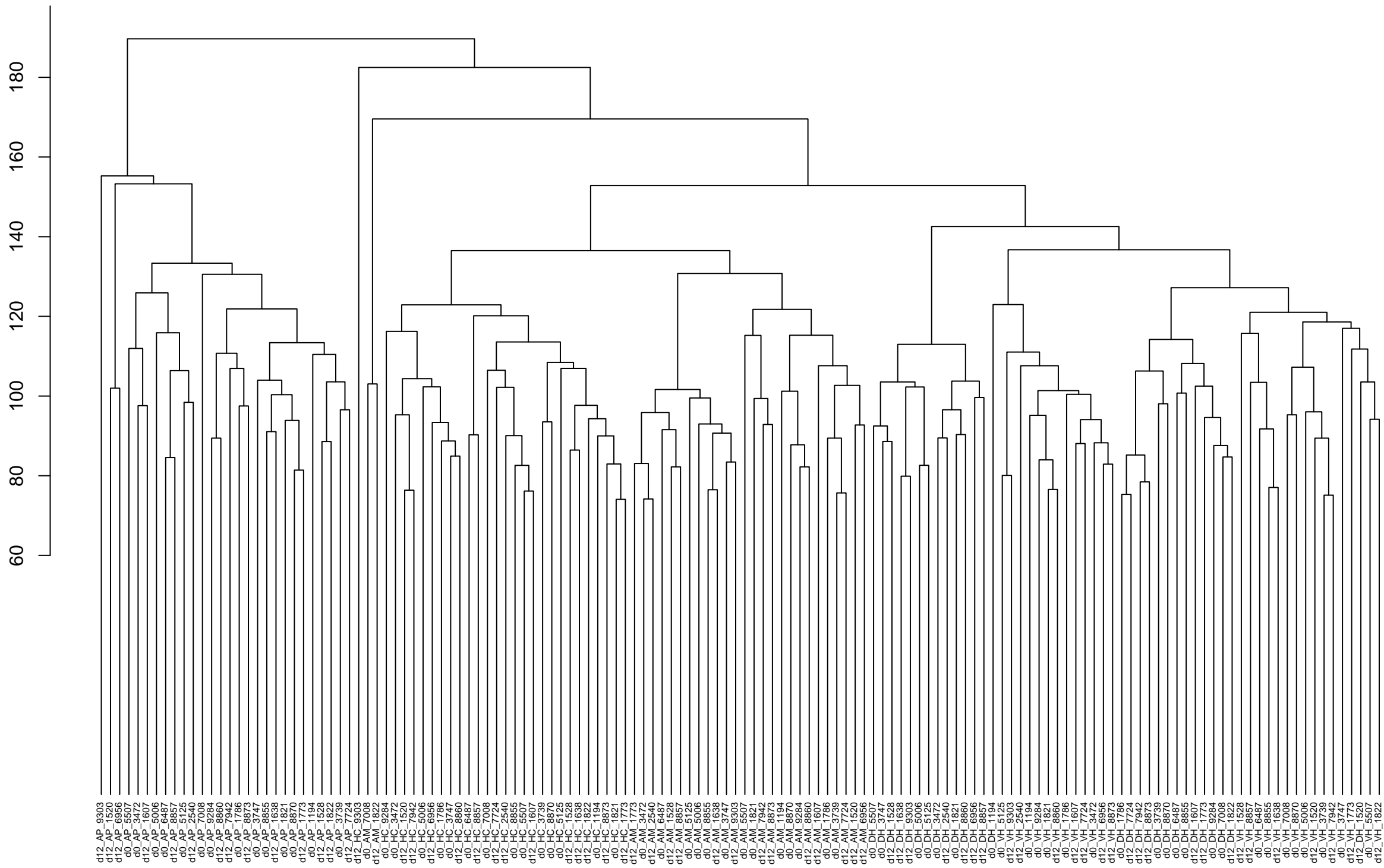

Supplement: Figure S1 — Hierarchical clustering of microarrays. Hierarchical cluster analysis of the gene expression intensities for all individuals showed that arrays from the same brain area tended to cluster together but the effect of day was not evident. (AP-Anterior Pituitary, AM-Amygdala, HC-Hippocampus, DH- Dorsal Hypothalamus, VH-Ventral Hypothalamus). (PDF) [file pone.0020413.s001.pdf]

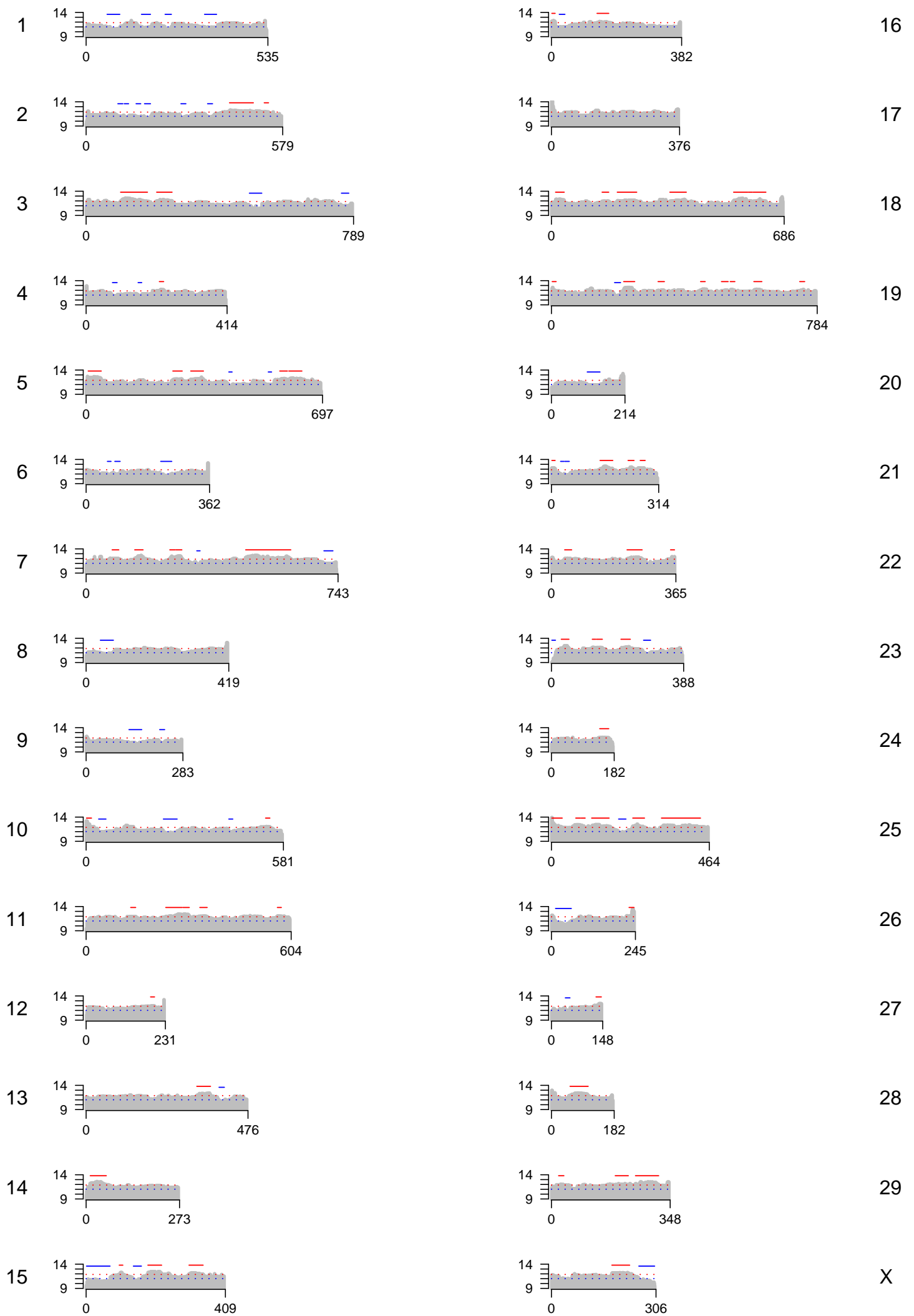

Supplement: Figure S2 — Chromosome wise transcriptome maps depicting the identified RIDGEs and anti-RIDGEs. The y-axis shows the median gene expression levels (log transformed normalised gene intensities ranging from 9 to 14) for the Ensembl genes on each chromosome represented on the array. The solid red and blue lines depict the RIDGEs and anti-RIDGEs respectively whereas the dotted red and blue lines represent the expression threshold to qualify as RIDGE or anti-RIDGE respectively. (PDF) [file pone.0020413.s002.pdf]

## Transcript length

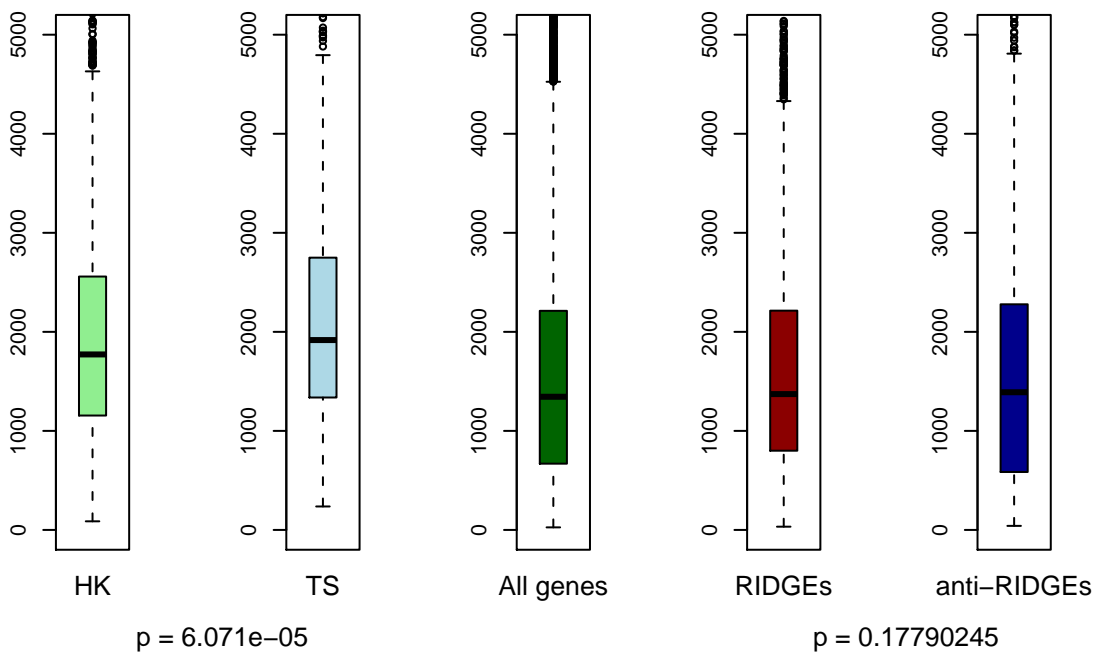

## Exon count

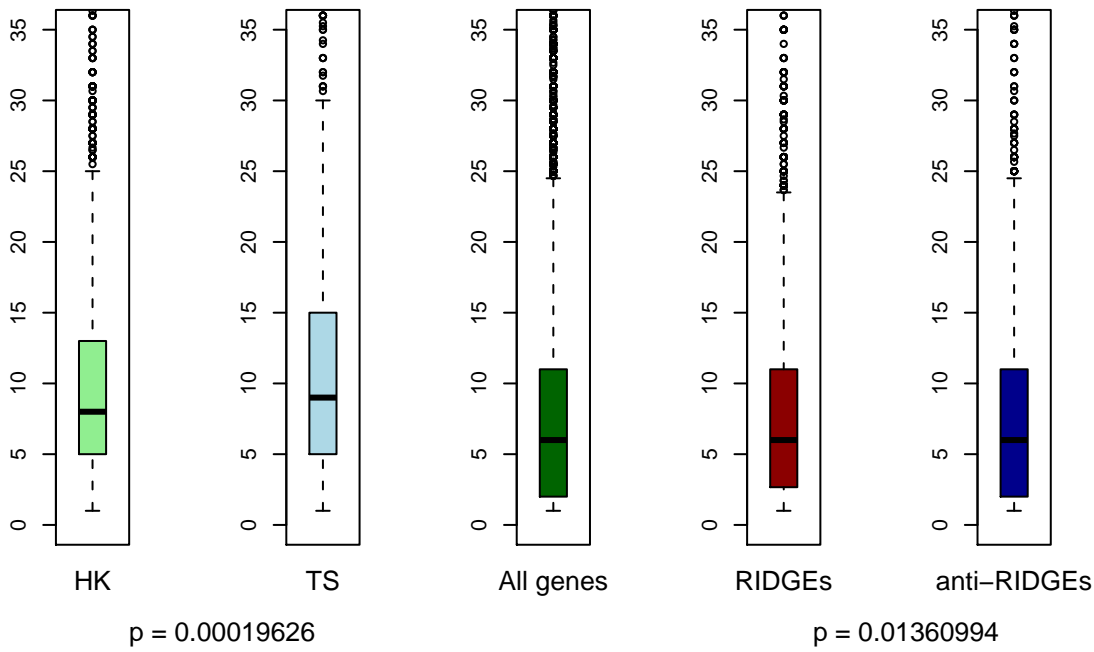

## Exon length

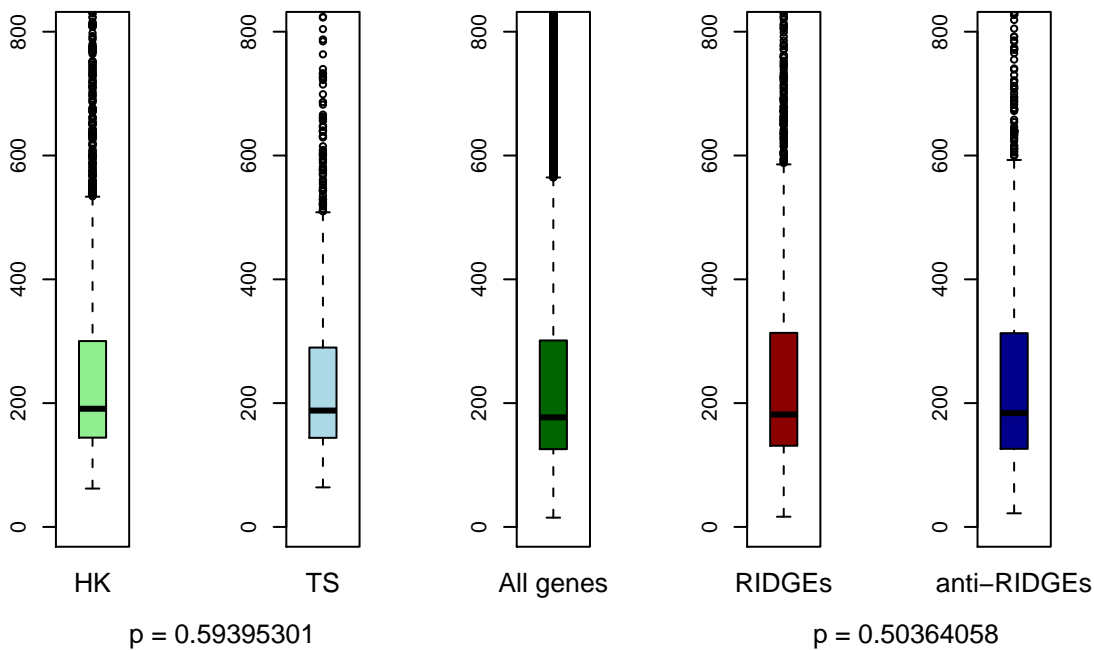

Supplement: Figure S3 — Genomic features of housekeeping vs. tissue-specific genes and of genes present on the RIDGEs vs. anti-RIDGEs. The following genomic features are represented here: Transcript length, exon count and exon length. The p-value of the significance of difference between the genomic feature comparison is given below each pair of boxplots separated by a boxplot depicting the feature for all genes together. The bottom and top of the box are represents the 25th and 75th percentile (the lower and upper quartiles, respectively), and the band near the middle of the box represents the 50th percentile (the median). The ends represent the lowest datum still within 1.5 IQR of the lower quartile, and the highest datum still within 1.5 IQR of the upper quartile. Any data not included between the ends are plotted as an outlier with a dot. (HK – housekeeping genes, TS – tissue-specific genes). (PDF) [file pone.0020413.s003.pdf]
